# Transcriptome analysis of stem development in the tumourous stem mustard *Brassica juncea *var. *tumida *Tsen et Lee by RNA sequencing

**DOI:** 10.1186/1471-2229-12-53

**Published:** 2012-04-21

**Authors:** Quan Sun, Guanfan Zhou, Yingfan Cai, Yonghong Fan, Xiaoyan Zhu, Yihua Liu, Xiaohong He, Jinjuan Shen, Huaizhong Jiang, Daiwen Hu, Zheng Pan, Liuxin Xiang, Guanghua He, Daiwen Dong, Jianping Yang

**Affiliations:** 1 College of Bioinformation, Chongqing University of Posts and Telecommunications, Chongqing 400065, China; 2 Institute of Chongqing Fuling Agricultural Sciences, Fuling 408000, China; 3 Chongqing Key Laboratory of Application and Safety Control of Genetically Modified Crops, Southwest University, Chongqing 400716, China

**Keywords:** Transcriptome, Tumourous stem mustard, Development, RNA sequencing, *Brassica juncea *var. *tumida*

## Abstract

**Background:**

Tumourous stem mustard (*Brassica juncea *var. *tumida *Tsen et Lee) is an economically and nutritionally important vegetable crop of the *Cruciferae *family that also provides the raw material for *Fuling *mustard. The genetics breeding, physiology, biochemistry and classification of mustards have been extensively studied, but little information is available on tumourous stem mustard at the molecular level. To gain greater insight into the molecular mechanisms underlying stem swelling in this vegetable and to provide additional information for molecular research and breeding, we sequenced the transcriptome of tumourous stem mustard at various stem developmental stages and compared it with that of a mutant variety lacking swollen stems.

**Results:**

Using Illumina short-read technology with a tag-based digital gene expression (DGE) system, we performed *de novo *transcriptome assembly and gene expression analysis. In our analysis, we assembled genetic information for tumourous stem mustard at various stem developmental stages. In addition, we constructed five DGE libraries, which covered the strains *Yong'an *and *Dayejie *at various development stages. Illumina sequencing identified 146,265 unigenes, including 11,245 clusters and 135,020 singletons. The unigenes were subjected to a BLAST search and annotated using the GO and KO databases. We also compared the gene expression profiles of three swollen stem samples with those of two non-swollen stem samples. A total of 1,042 genes with significantly different expression levels occurring simultaneously in the six comparison groups were screened out. Finally, the altered expression levels of a number of randomly selected genes were confirmed by quantitative real-time PCR.

**Conclusions:**

Our data provide comprehensive gene expression information at the transcriptional level and the first insight into the understanding of the molecular mechanisms and regulatory pathways of stem swelling and development in this plant, and will help define new mechanisms of stem development in non-model plant organisms.

## Background

The tumourous stem mustard, *Brassica juncea *(*Cruciferae*), is an important vegetable and the raw material for *Fuling *mustard. The genetics breeding, physiology, biochemistry and classification of mustards have been extensively studied, but little work has been done at the molecular level. The three diploid *(B. nigra, B. oleracea*, and *B. campestris*) and three allotetraploid *(B. carinata, B. juncea*, and *B. napus*) species of *Brassica*, related according to the U-triangle theory, are one of the best model systems to study polyploidy and genetic relationships [1]. Thus, investigating tumourous stem mustard at the molecular level may help to clarify the genetic relationships mentioned above.

Development of the stem in tumourous stem mustard, which is pickled or eaten fresh, is directly related to the quality and yield of tumourous mustards. Stem swelling is a key characteristic of tumourous stem formation, and although many new cultivars of tumourous stem mustard have been bred, the molecular mechanism underlying stem swelling is unclear. Tumourous stem mustard is an annual plant, and the stem does not swell except in plants sown between mid-September and mid-October in Chongqing and the other valleys of the Yangtze River, China; thus, the production period of edible stems is limited [2,3]. Identification of the genes controlling stem swelling and the regulatory network would facilitate molecular breeding and increase the yield and quality of this crop.

Tumourous stem mustard shares a close genetic relationship with the model plants *Arabidopsis thaliana *and *Arabidopsis lyrata*, whose genetic backgrounds are more clearly understood. Thus, *A. thaliana *may aid in our understanding of the mechanism of swelling in tumourous stem mustard. In recent years, novel, high-throughput, deep sequencing technologies have allowed the efficient generation of large-scale ESTs and improved the speed of gene discovery [4]. In addition, *A. thaliana *microarrays may be used to study stem swelling because of the close genetic relationship between that organism and tumourous stem mustard. RNA sequencing (RNA-Seq) generates absolute rather than relative gene expression measurements and provides greater insight and accuracy than microarrays [5-7].

In this study, we generated over 5 billion bases of high-quality DNA sequence using Illumina technology and demonstrated the suitability of short-read sequencing for the *de novo *assembly and annotation of genes expressed without prior genome information. In addition, we constructed five digital gene expression (DGE) libraries and compared the gene expression profiles of tumourous stem mustard at different developmental stages with those of different varieties. The assembled annotated transcriptome sequences and gene expression profiles will facilitate the identification of genes involved in tumourous stem mustard swelling and be a useful reference for other *Cruciferae *developmental studies.

## Methods

### Plant material

The tumourous stem mustard (*Brassica juncea *var. *tumida *Tsen et Lee) strains *Yong'an *(with inflated tumourous stems) and a mutant variety *Dayejie *(without inflated stems) were collected from the Chongqing Fuling Institute of Agricultural Sciences, Chongqing, China. After seeding on October 5th, *Dayejie *and four developmental stages of *Yong'an *were collected in February of the next year. *Dayejie *stems were collected 22 weeks after seeding (*daye3bianzhong*). The stems of *Yong'an *were collected 18, 20, 22, and 25 weeks after seeding (respectively: *yongan1hao*, uninflated; *yongan2hao*, one week before the start of inflation; *yongan3hao*, one week after the start of inflation; and *yongan4hao*, one month after the start of inflation). After washing in physiological saline and 0.1% DEPC-treated water, the fresh samples were stored in liquid nitrogen until the extraction of total RNA.

### cDNA preparation and Illumina sequencing

Total RNA was extracted using a Plant RNA Kit according to the manufacturer's instructions (Watson Biotech, Shanghai, China). The quantity of RNA was verified by an ultraviolet spectrometer and electrophoresis on a denaturing formaldehyde agarose gel. To obtain complete gene expression information, a pooled RNA sample from tissues of different developmental stages (stems of the yongan1hao, yongan2hao, yongan3hao, and yongan4hao stages) was used for transcriptome analysis. Oligo(dT) beads were used to isolate poly(A) + mRNA from the total RNA. Fragmentation buffer was added to disrupt the mRNA into short fragments. Taking these short fragments as templates, a random hexamer primer was used to synthesise first-strand cDNA. Second-strand cDNA was synthesised using buffer, dNTPs, RNase, and DNA polymerase I. The resulting short fragments were purified with a QiaQuick PCR extraction kit and resolved with EB buffer for end repair and addition of a poly(A) tail. Afterwards, the short fragments were connected with sequencing adapters. Following agarose gel electrophoresis, suitable fragments were selected as templates for PCR. Finally, the library was sequenced using Illumina HiSeq™ 2000.

### Transcriptome analysis

The raw data from the images were collected using Solexa GA pipeline 1.6 by removing low-quality reads (reads with unknown sequences 'N'), adaptor sequence fragments, and empty reads. Next, *de novo *assembly of the transcriptome into unigenes was carried out with SOAPdenovo, a short-reads assembly program [8]. Briefly, SOAPdenovo first combines reads with a particular overlap to form longer fragments without N, which are called contigs. Then, the reads are mapped back to contigs. The program is able to detect contigs from the same transcript as well as the sequences between these contigs using paired-end reads. Next, SOAPdenovo connects the contigs using N to represent unknown sequences between each two contigs, and scaffolds are made. Paired-end reads are used again for gap filling of the scaffolds to obtain sequences with the lowest Ns and which cannot be extended on either end. Such sequences are defined as unigenes.

Subsequently, a BLASTX alignment (e-value < 0.00001) was performed between the unigenes and protein databases, including the non-redundant (nr), Swiss-Prot, Kyoto Encyclopaedia of Genes and Genomes (KEGG), and COG databases, and the best alignments were used to decide the sequence direction of the unigenes. If the results from the different databases conflicted, a priority order of nr, Swiss-Prot, KEGG, and COG was followed to decide the sequence direction of the unigenes. When a unigene happened to be unaligned in any of the above databases, ESTScan was used to predict its coding regions and decide its sequence direction [9]. In the final step, using nr annotation, the Blast2GO program was used to obtain the Gene Ontology (GO) and KEGG annotations of the unigenes [10]. After obtaining the GO annotation for each unigene, WEGO software was used to classify the unigenes by function and to determine the distribution of gene functions in the species at the macro level [11]. All raw transcriptome data have been deposited at the sequence read archive (SRA) of NCBI.

### DGE library preparation and sequencing

Total RNA was extracted from the stems of yongan1hao, yongan2hao, yongan3hao, yongan4hao, and daye3bianzhong using a Plant RNA Kit (Watson Biotech) according to the manufacturer's instructions. Next, a DGE library was prepared using an Illumina gene expression sample prep kit. Briefly, total RNA from the samples was used for mRNA capture with magnetic oligo(dT) beads. Double-stranded cDNAs were synthesised directly on the poly(A) + RNA-bound beads and then digested with *Nla*III. Those cDNA fragments with 3' ends were purified from the magnetic beads, and Illumina adaptor 1 was added to their 5' ends. After digestion with *Mme*I, which recognises the junction between Illumina adaptor 1 and the sequence CATG, 21-bp tags containing adaptor 2 were ligated to the 3' ends of the tags to create a tag library. The library was amplified by PCR over 15 cycles, and 85-bp strips were purified by PAGE. The single-stranded molecules were attached to the Illumina chip for sequencing. All raw tag data were deposited at the SRA of NCBI.

### DGE analysis

The raw image data were transformed by base calling into sequence data. To map the DGE tags, the sequenced raw data were filtered to remove adaptor sequences, low-quality sequences (tags with unknown sequences), empty tags (no tag sequence between the adaptors), and tags with only one copy number (probable sequencing error). For tag annotation, clean tags containing CATG and the 21-bp tag sequences were mapped to our transcriptome reference database, allowing no more than one nucleotide mismatch. The clean tags were designated as unambiguous clean tags. For gene expression analysis, the number of expressed tags was calculated and normalised to the number of transcripts per million tags.

To compare the differences in gene expression, the tag frequency in each DGE library was statistically analysed according to the method of Audic and Claverie [12]. We used a false discovery rate of < 0.001 and an absolute value of the log 2 ratio of > 1 as the threshold for judging the significance of the gene expression differences. Next, the differentially expressed genes were subjected to GO and KEGG Ontology (KO) enrichment analysis. Enriched p-values were calculated according to the hypergeometric test:

$$\text{P}=1-\overset{m-1}{\underset{i=0}{\sum }}\frac{\left(\begin{array}{c} M\\ i \end{array}\right)\left(\begin{array}{c} N-M\\ n-i \end{array}\right)}{\left(\begin{array}{c} N\\ n \end{array}\right)}$$

In this equation, *N *represents the number of genes with a GO/KO annotation, *n *represents the number of differentially expressed genes in *N, M *represents the number of genes in each GO/KO term, and *m *represents the number of differentially expressed genes in each GO/KO term. For GO enrichment analysis, all of the p-values were subjected to Bonferroni correction. A corrected p-value of < 0.05 was selected as the threshold for determining significant enrichment of the gene sets.

### Quantitative real-time PCR (qRT-PCR) validation

Total RNA was extracted as described for DGE library preparation and sequencing. Total RNA (1 μg) from each sample was reverse-transcribed in a 10-μl reaction using the AMV RNA PCR Kit 3.0 (Takara). The sequences of the primers used are given in Additional file 1: Table S1. The 18 s rRNA gene of tumourous stem mustard was used as an internal control. qRT-PCR was performed using SYBR Premix Ex Taq™ Kit (Takara) according to the manufacturer's protocol. The selected genes were verified using a Bio-Rad iQ5 real-time PCR detection system with a cycling temperature of 57°C and with a single peak on the melting curve to ensure a single product. At least three replicates were tested per sample.

## Results

### Illumina sequencing and sequence assembly

**Figure 1:**
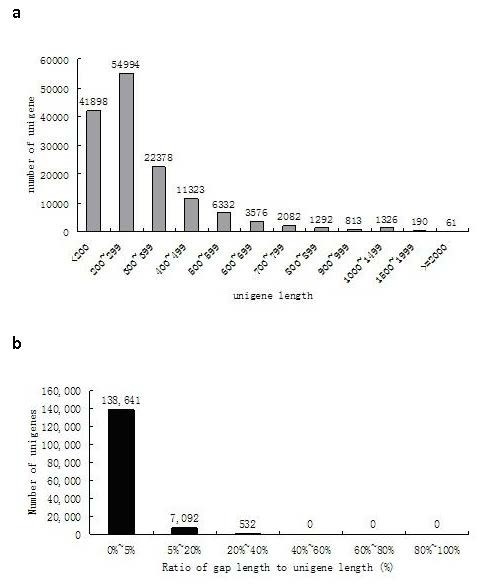
**Unigene size and gaps distribution**.

A total of 54,577,780 reads (accumulated length, 4,912,000,200 bp; SRA accession number SRX108497) were generated through Illumina sequencing and assembled into 712,909 contigs. Using paired end joining and gap filling, the contigs were further assembled into 247,432 scaffolds with a mean length of 232 bp. After clustering the scaffolds together with the nucleotide sequences available from the NCBI, we obtained 146,265 unigenes, including 11,245 clusters and 135,020 singletons, with a mean length of 304 bp. The sequence clusters had two to seven scaffolds per cluster, and 92% of the clusters contained only one scaffold. The size distribution indicated that the lengths of the 1,577 unigenes exceeded 1,000 bp (Figure 1a).

To evaluate the quality of the dataset, the ratio of the gap length to the length of the assembled unigenes was analysed (Figure 1b). Most of the unigenes showed gap lengths < 5% of the total length, which accounted for 94.79% of the total number of unigenes (146,265).

### Annotation of predicted proteins

**Figure 2:**
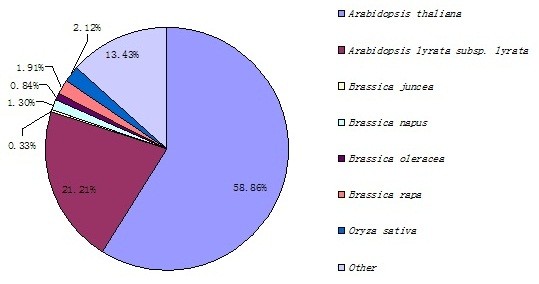
**Species distribution of the Blastx results**.

To annotate the unigenes, we first searched the reference sequences using BLASTX against the nr NCBI protein database with a cut-off e-value of 10^-5^. A total of 105,555 unigenes (72% of all unigenes) returned a significant BLAST result (shown in Additional file 2: Table S2). The species distribution of the best match result for each sequence is shown in Figure 2. The sequences had a 58.86% match with *A. thaliana*, followed by *Arabidopsis lyrata *subsp. *lyrata *(21.21%), *Brassica *(4.38%), and *Oryza sativa *(2.12%).

### COG classification

**Figure 3:**
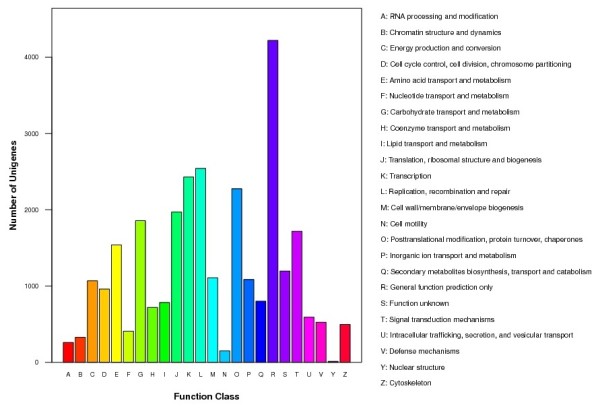
**COG Function Classification of the Stem tumor mustard transcriptome**. A total of 29,044 unigenes showing significant homology to the COGs database at NCBI (E-vaule < = 1.0e^-5^) have a COG classification among the 24 cateories.

The assembled unigenes were compared against the COG database for the analysis of phylogenetically widespread domain families. The results revealed 29,044 unigenes with significant homology and assigned them to the appropriate COG clusters. These COG classifications were grouped into 24 functional categories (Figure 3). The five largest categories were 'general function' (14.5%); 'replication, recombination, and repair' (8.7%); 'transcription' (8.4%); 'translation, ribosomal structure, and biogenesis' (6.8%); and 'carbohydrate transport and metabolism' (6.4%).

### GO classification

**Figure 4:**
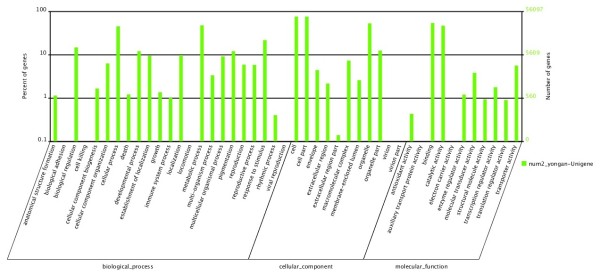
**Gene Ontology Classification of the Stem tumor mustard transcriptome**.

GO assignments were used to classify the functions of the predicted tumourous stem mustard genes. Based on sequence homology, 56,098 sequences were categorised into 45 functional groups (Figure 4). In each of the three main categories (biological process, cellular component, and molecular function) of the GO classification, the major subcategories were as follows: six subcategories for biological process ('biological regulation', 'cellular process', 'developmental process', 'metabolic process', 'pigmentation' and 'response to stimulus'); four subcategories for cellular component ('cell', 'cell part', 'organelle' and 'organelle part'); and two subcategories for molecular function ('binding' and 'catalytic activity'). Only a few genes were clustered in terms of 'biological adhesion', 'cell killing', 'locomotion', viral reproduction', 'virion', 'virion part', 'auxiliary transport protein activity' and 'electron carrier activity'. Figure 4 shows the GO classification of the tumourous stem mustard transcriptome.

### Functional classification using the KEGG database

Functional classification and pathway assignment was performed using the KEGG database [13]. First, the 146,265 unigenes with an e-value ≤ 1e^-05 ^were compared using BLASTX against the KEGG database. In total, 39,203 unigenes were assigned to 119 KEGG pathways (see Additional file 3: Table S3). The major pathways were 'metabolic pathways [ko01100]', 'biosynthesis of secondary metabolites [ko01110]', 'plant-pathogen interaction [ko04626]', 'spliceosome [ko03040]' and 'starch and sucrose metabolism [ko00500]'; the gene numbers and percentages assigned to these pathways were 8,939 (22.8%), 4,470 (12.17%), 2,792 (7.12%), 1,786 (4.56%) and 1,180 (3.01%), respectively.

### DGE library sequencing

Five DGE libraries for tumourous stem mustard were sequenced (daye3bianzhong, yongan1hao, yongan2hao, yongan3hao, and yongan4hao, corresponding to the SRA accession numbers SRX108496, SRX108498, SRX108499, SRX108500, and SRX108501, respectively), which generated approximately 11 to 12 million high-quality reads for each library (Table 1). The percentage of clean reads among the raw reads in each library was > 97% (Table 2). Among the clean reads, the number of sequences that could be mapped to unigenes ranged from 2.3 to 2.9 million, and the percentage of cleans reads ranged from 26.75 to 31.36% in the five libraries. As Table 1 shows, the vast majority of these mapped reads were uniquely matched to unigenes, and the percentage of multi-position matched reads was no more than 0.31%.

**Table 1:** Statistics of DGE sequencing

Summary	Daye3bianzhong	Yongan1hao	Yongan2hao	Yongan3hao	Yongan4hao
Total Reads	11769284	11889409	11111041	12447409	12067757
Total Mapped Reads	3148437(26.75%)	3427726(28.83%)	2946873(26.52%)	3749087(30.12%)	3784412(31.36%)
perfect match	2340718(19.89%)	2743445(23.07%)	2344512(21.1%)	2934783(23.58%)	2872592(23.8%)
< = 2 bp mismatch	807719(6.86%)	684281(5.76%)	602361(5.42%)	814304(6.54%)	911820(7.56%)
unique match	3116463(26.48%)	3390730(28.52%)	2924115(26.32%)	3733720(30%)	3767740(31.22%)
multi-position match	31974(0.27%)	36996(0.31%)	22758(0.2%)	15367(0.12%)	16672(0.14%)
Total Unmapped Reads	8620847(73.25%)	8461683(71.17%)	8164168(73.48%)	8698322(69.88%)	8283345(68.64%)

**Table 2:** Different components of the raw reads in each sample

Summary	Clean reads	Only Adaptor	Containing N	Low Quality
Daye3bianzhong	11769284(98.64%)	73363(0.61%)	159(0.00%)	88436(0.74%)
Yongan1hao	11889409(98.69%	84548(0.70%)	192(0.00%)	73571(0.61)
Yongan2hao	11111041(97.83)	179199(1.58%)	177(0.00%)	66890(0.59%)
Yongan3hao	12447409(98.85%)	83532(0.66%)	204(0.00%)	61217(0.49%)
Yongan4hao	12067757(98.86%)	85102(0.70%)	259(0.00%)	54189(0.44%)

Note: The percentages of reads containing N, adaptors, low quality, clean reads. The numbers in parentheses indicate the percentage of each type of read among the total raw reads

### Gene expression variations among the different samples

**Figure 5:**
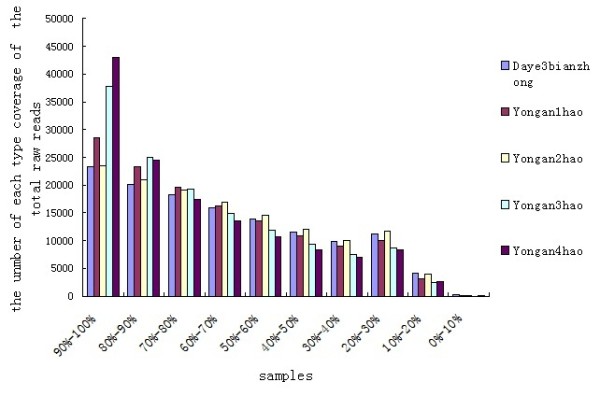
**Distribution of reference unigenes' coverage in each sample**.

First, to evaluate the DGE data, we analysed the distribution of unigene coverage in each sample, which is the number of clean reads that aligned to the reference unigenes. As shown in Figure 5, most unigene coverage was > 50% (dayebianzhong, 70% of all unigenes; yongan1hao, 75% of all unigenes; yongan2hao, 78% of all unigenes; yongan3hao, 72% of all unigenes; and yongan4hao, 80% of all unigenes). Second, the number of clean reads was calculated and the gene expression level was calculated using the reads per kb per million reads method for each unigene (shown in Additional file 4: Table S4) [7]. Third, the differentially expressed genes were identified using an algorithm developed by Audic et al. [12] at different developmental stages and in various samples.

**Figure 6:**
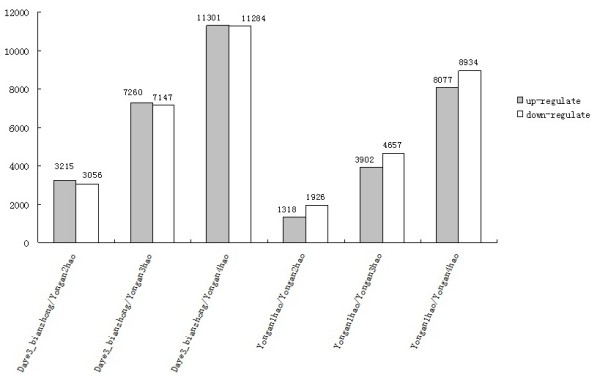
**Numbers of DGE unigenes in each comparison**. The numbers on column showed quantity of up-(gray) and down-(blank) unigenes. The results of six comparisons are shown.

Variations in gene expression were identified based on comparisons of daye3bianzhong and yongan2hao to yongan4hao, and yongan1hao and yongan2hao to yongan4hao. The results showed significantly differentially expressed genes in the above comparisons: (a) between daya3bianzhong and yongan2hao, 3,215 and 3,056 genes were up- and downregulated, respectively; (b) between daya3bianzhong and yongan3hao, 7,260 and 7,147 genes were up- and downregulated, respectively; (c) between daya3bianzhong and yongan4hao, 11,301 and 11,284 genes were up- and downregulated, respectively; (d) between yongan1hao and yongan2hao, 1,318 and 1,926 genes were up- and downregulated, respectively; (e) between yongan1hao and yongan3hao, 3,902 and 4,657 genes were up- and downregulated, respectively; and (f) between yongan1hao and yongan4hao, 8,077 and 8,934 genes were up- and downregulated, respectively (Figure 6).

**Figure 7:**
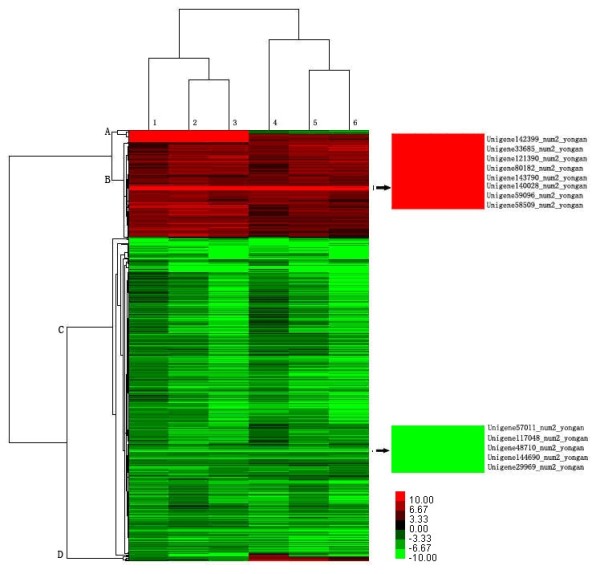
**Clustering of selected out genes expression profiles at 6 different comparison**. Expression ratios are expressed as log 2 values. Number 1 to 6 indicated expression ratios of yongan2hao/daye3bianzhong, yongan3hao/daye3bianzhong, yongan4hao/daye3bianzhong, yongan2hao/yongan1hao, yongan3hao/yongan1hao, yongan4hao/yongan1hao, respectively. Red color represents increasing level of the gene expression and green color indicates decreasion of the gene expression after challenging with control samples. Arrow showed the genes that expression ratios absolute values > 10 among six comparison groups.

The 1,042 genes occurring simultaneously in the six comparisons above (a-f) were screened out, and the comparison ratio values of these genes were used to build a cluster tree. As Figure 7 shows, the genes were divided into four groups based on whether they showed a trend toward up- (red) or downregulation (green). To investigate some of the most differentially up- and downregulated genes (absolute ratios of log 2 value > 10 in six comparison groups; shown by the arrow in Figure 7), eight up-regulated and five downregulated genes clustered and were screened out. Of these thirteen genes, only one downregulated gene had a defined function (Unigene144690_num2_yongan was similar to NLI-interacting factor family protein), wherea five upregulated genes had defined functions (Unigene142399_num2_yongan was similar to phytochrome kinase substrate 1, Unigene121390_num2_yongan was similar to sulfotransferase family protein, Unigene140028_num2_yongan was similar to ethylene-responsive transcription factor ABR1, Unigene59096_num2_yongan was similar to reverse transcriptase, and Unigene58509_num2_yongan was similar to mucin-like protein). A total of seven genes among the thirteen have unknown functions (see Additional file 5: Table S5).

**Figure 8:**
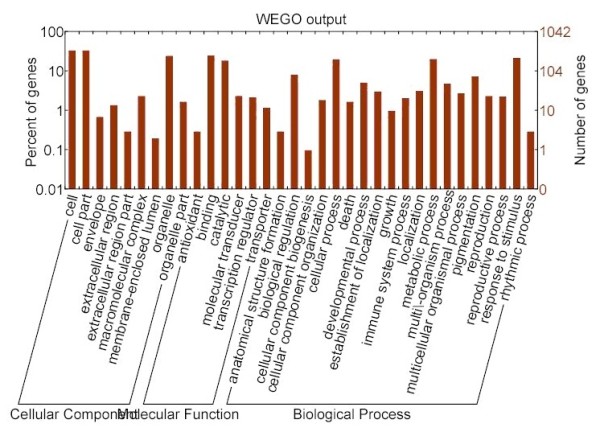
**GO categories of the selected genes**.

Next, the 1,042 screened genes were analysed for function using the GO annotation system. The major subcategories were as follows: three subcategories ('cell', 'cell part' and 'organelle') in the cellular component cluster; two subcategories ('binding' and 'catalytic activity') in the molecular function cluster; and three subcategories ('cellular process', 'metabolic process' and 'response to stimulus') in the biological process cluster (Figure 8). The KO categories were further analysed, and the results indicated that the major pathways were 'metabolic pathways [ko01100]', 'biosynthesis of secondary metabolites [ko01110]', 'plant hormone signal transduction [ko04626]' and 'microbial metabolism in diverse environments [ko01120]' (see Additional file 6: Table S6 and the KO annotation of the 1,024 genes in Additional file 7: Table S7).

### Validation of RNA-Seq-based gene expression

**Figure 9:**
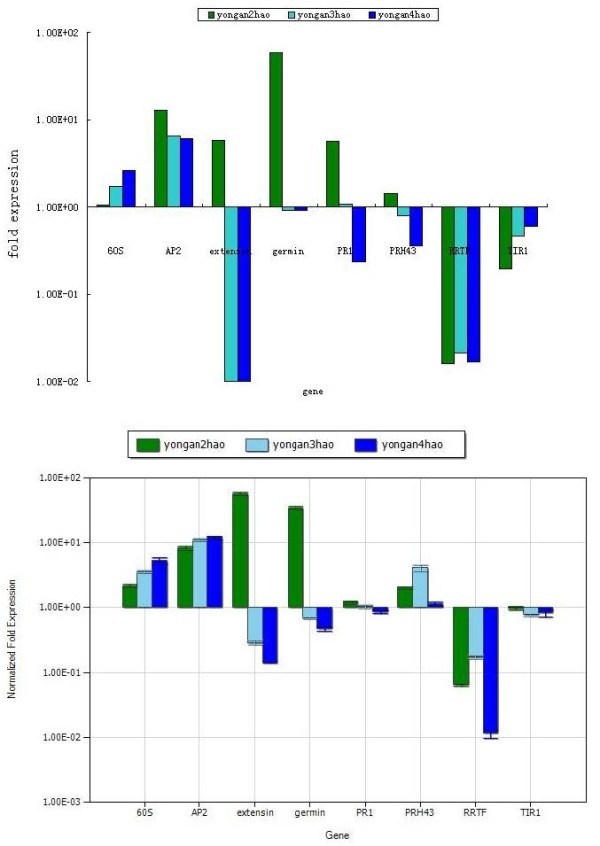
**Expression pattern of random selected genes**. (A) Gene expression data for DGE analysis. The fold changes of the genes were calculated as the log2 vaule of yongan2hao/yongan1hao (the cutline of yongan2hao), yongan3hao/yongan1hao (the cutline of yongan3hao) and yongan4hao/yongan1hao (the cutline of yongan4hao) comparison and shown on the y-axis.(B) The qRT-PCR analysis of gene expression data. Expression ratios of these genes in yongan2hao, yongan3hao and yongan4hao were compared to yongan1hao, respectively.

To validate the expression profiles obtained by RNA-Seq, real-time RT-PCR was performed on eight genes selected at random with high or low expression levels. Expression comparisons were performed between yongan2hao and yongan1hao, yongan3hao and yongan1hao, and yongan4hao and yongan1hao by qRT-PCR. For all of the genes, the trend in real-time RT-PCR expression was in agreement with the RNA-Seq data except for PRH43 (Figure 9).

## Discussion

Tumourous stem mustard, an important cash crop and the raw material for *Fuling *pickles, is a world-famous vegetable crop. Presently, many varieties of tumourous stem mustard have been bred, but the regulatory pathway and molecular mechanism of mustard tumourous stem development are unclear, and molecular biological research on tumourous stem mustard is rare.

By transcriptome sequence analysis, we obtained 54,577,780 reads corresponding to about 5 Gb of raw sequence data. The predicted 146,265 unigenes were subjected to BLAST annotation, and 72% of the unigenes returned a significant BLAST result. As expected, most unigenes shared the highest sequence similarity with crucifers (*A. thaliana, A. lyrata *subsp. *lyrata*, and *Brassica*). The number of genes similar to *Brassica *genes was lower than the number of similar genes between *A. thaliana *and *A. lyrata *subsp. *lyrata *(Figure 2), possibly due to the characteristics of the *B. juncea *transcriptome in the NCBI protein database. *Arabidopsis thaliana *is an important model plant with a clear genetic background that is very useful for researching gene functions in tumourous stem mustard. Our transcriptome analysis is the first high-throughput sequencing of tumourous stem mustard and will serve as a basis for other studies.

To investigate the regulatory pathway and molecular mechanism of tumour swelling, we created five DGE libraries from plants at different developmental stages and samples from a non-swollen mutant to analyse the gene expression patterns at various developmental stages. The quality of the DGE libraries was further confirmed by qRT-PCR analysis. Because the tumours continue to swell for a long period after initiation, different swelling stages were selected for each experimental group, and non-swollen stages of *Yong'an *and the non-swollen mutant strain *Dayejie *were used for comparison. The DGE profiles of the swelling stages were compared with the controls. Different genes may be involved in tumourous stem formation, and the common genes in the six comparison groups reduced the number of differentially expressed genes that might be related to tumourous stem development.

Compared with the GO annotation results of the DGE screening of genes in the transcriptome data, we found no genes distributed in the three molecular function subcategories (enzyme regulator activity, structural molecular activity, or translation regulator activity) in the DGE group, indicating that these three subcategories are not related to tumour swelling. A comparison of the results of Additional file 3: Table S3 and Table S6 showed that the pathway order number of 'Plant hormone signal transduction' was about 10 (Additional file 6: Table S6), suggesting that these genes were screened out and that the pathway 'plant hormone signal transduction' is related to tumour swelling. Mapping the DGE data back to the transcriptome database revealed that about 30% of the reads were mapped and that > 60% remained transcribed sequences. Although large amounts of data were obtained by transcriptome sequencing, the reference sequences may still be insufficient and may have caused the lower mapped ratio, which could be resolved by increasing the sequencing depth and enhancing the accuracy of the assembly.

Although 1,042 differentially expressed genes were discovered using the above method, the key genes related to tumourous stem formation need to be analysed further. The genes with the greatest changes in expression were selected for further study; a log 2 ratio value > 10 was used as a threshold to select thirteen genes for further analysis. Seven of the genes have unknown functions and six genes have a functional annotation based on sequence similarity. Of the six annotated genes, four gene functions require further clarification, and two genes, phytochrome kinase substrate 1 (PKS1) (Unigene142399_num2_yongan) and ABR1 (ABA REPRESSOR1) (Unigene140028_num2_yongan), have functions whose details are relatively well known. Girdhar et al. [14] determined that ABR1 functions in the negative regulation of ABA responses during seed germination and plays a role in the ABA signalling pathway in *Arabidopsis*. In this study, the expression level of *ABR1 *was significantly higher in the inflation stage of the stem tumour. This suggests that ABA signalling is related to stem inflation. As Additional file 6: Table S6 shows, four genes may be involved in the 'plant hormone signal transduction [ko04075]' pathway, suggesting that plant hormones are related to stem swelling; however, *ABR1 *was not one of the four genes annotated in the 'plant hormone signal transduction' pathway in this study. During the inflation stage of stem tumours, plant hormones play key roles in the number of cells in the stem, which increase rapidly accompanied by cell splintering. *PKS1 *expression was upregulated during the tumour inflation stage. To our knowledge, PKS1 is phosphorylated in a phytochrome-dependent manner and negatively regulates phytochrome light signalling in *Arabidopsis *[15]. Other reports have shown that PKS1 regulates root phototropism and gravitropism and leaf flattening and positioning, and that it affects the state of phytochrome A in etiolated *Arabidopsis *seedlings [16-20]. Chen et al. [21,22] found that illumination and temperature were the main factors affecting the formation of stem tumours, but they did not determine whether PKS1 plays key roles in illumination. Is PKS1 related to stem inflation in tumourous stem mustard? Whether or not the overexpression or knockout/down of ABR1 and PKS1 alters the trend of inflation requires further research.

## Conclusions

Although the molecular functions of individual tumourous mustard genes and the associated signal transduction pathways remain largely unknown, the present transcriptome analysis provides valuable information regarding tumourous stem development, which may facilitate future investigations of the detailed regulatory mechanisms and pathways. Additionally, we obtained about 5 Gb of raw sequence data and predicted 146,265 assembled unigenes, and annotated 105,555 unigenes using Illumina sequencing technology. This is the first large-scale transcriptome and expression study of the tumourous stem mustard, *Brassica juncea *var. *tumida *Tsen et Lee for which the data has been deposited in GenBank (as of October 2011). We believe that our data not only provide more molecular information for researchers, but will also help to accelerate gene expression and function research in *Brassica juncea *as well as that on the evolutionary relationships of the *Cruciferae*.

## Authors' contributions

CYF conceived the study. CYF, SQ, ZXY participated in experiment materials preparation. ZXY participated in RNA extraction. SQ analyzed data and performed qRT-PCR materials. CYF, SQ wrote the paper. ZGF provided the plant varieties and participated in experiment materials preparation, FYH, LYH, HXH, SJJ, JHZ, HDW, PZ, XLX and YJP participated in part of experiment. All authors read and approved the final manuscript.

## Supplementary Material

Additional file 1 **Table S1 Primers used in qRT-PCR for validating differentially expressed genes**. Click here for file

Additional file 2 **Table S2 Top hits obtained by Blastx for the unigenes**. Blastx against the nr, Swiss-Prot, KEGG and COG protein database was used with a cutoff E-value < 1e-5 (part). Click here for file

Additional file 3 **Table S3 KO's assigned of unigenes**. Click here for file

Additional file 4 **Table S4 Expression level of unigenes in each library (part)**. Click here for file

Additional file 5 **Table S5 Discription of thirteen screened out genes**. Expression ratios absolute values > 10 in six comparison groups were used for rule. Click here for file

Additional file 6 **Table S6 KO's assigned of screening 1024 different genes**. Click here for file

Additional file 7 **Table S7 The unigenes' ID of screened out genes**. Click here for file
