# Correction: Transcriptome analysis of stem development in the tumourous stem mustard *Brassica juncea* var. *tumida* Tsen et Lee by RNA sequencing

**DOI:** 10.1186/1471-2229-13-90

**Published:** 2013-06-17

**Authors:** Quan Sun, Guanfan Zhou, Yingfan Cai, Yonghong Fan, Xiaoyan Zhu, Yihua Liu, Xiaohong He, Jinjuan Shen, Huaizhong Jiang, Daiwen Hu, Zheng Pan, Liuxin Xiang, Guanghua He, Daiwen Dong, Jianping Yang

**Affiliations:** 1 College of Bioinformation, Chongqing University of Posts and Telecommunications, Chongqing 400065, China; 2 Institute of Chongqing Fuling Agricultural Sciences, Fuling 408000, China; 3 Chongqing Key Laboratory of Application and Safety Control of Genetically Modified Crops, Southwest University, Chongqing 400716, China

## Correction

After the publication of our study [1], we became aware of errors in the proofreading of the article that caused a description mistake in the experimental methods section. These mistakes do not affect the final information in the analysis and results.

Specifically, the following amendments to the Methods section should be noted:

1. DGE library preparation and sequencing. Methods section paragraph 9, lines 3–11 should read:

Next, the DGE library was prepared using same method as above transcriptome library construction except the fragments about 200 were selected as templates for PCR. Finally, the library was sequenced using Illumina HiSeq™ 2000. All raw read data has been deposited in SRA (NCBI).

2. DGE analysis, paragraph 10 should read:

‘The raw image data were transformed by base calling into sequence data. To map the DGE reads, the sequenced raw data were filtered to remove adaptor sequences, low-quality sequences (reads with unknown sequences ‘N’), empty reads. For reads annotation, clean reads sequences were mapped to our transcriptome reference database, allowing no more than two nucleotide mismatch. For gene expression analysis, the number of expressed sequence was calculated and normalised to the number of Reads Per Kb per Million reads (RPKM).’

3. DGE analysis, paragraph 10, lines 1–2 should read:

‘To compare the differences in gene expression, the read frequency in each DGE library was statistically analysed according to the method of Audic and Claverie’

We apologize for any inconvenience that this may have caused to the readers.
